# Las noticias de Madrid (News from Madrid)

**DOI:** 10.1186/1750-1326-1-10

**Published:** 2006-09-06

**Authors:** Suzanne Elizabeth Wahrle

**Affiliations:** 1 Department of Neurology, Washington University School of Medicine. Campus Box 8111, 660 S. Euclid Ave., St. Louis, MO 63110, USA

## Abstract

Over 5,000 participants attended the 10th International Conference on Alzheimer's Disease (ICAD) and Related Disorders in Madrid, Spain from July 15–20, 2006. Highlights of the conference included reports on brain imaging, the discovery of mutations in the progranulin gene that cause frontotemporal dementia, the finding that neuregulin-1 is a substrate for BACE1 and new interest in the connection between Alzheimer's disease and metabolic syndromes.

## 

The 10^th ^International Conference on Alzheimer's Disease (ICAD) and Related Disorders was held in the Spanish capitol of Madrid from July 15–20, 2006. More than 5,000 researchers, students and clinicians came to Madrid to present their research and learn about the findings of others. The welcome reception at Palacio Negralejo, a sprawling estate on the outskirts of Madrid, allowed attendees to meet collaborators and friends and sample some typical Spanish food: Iberic ham, seafood paella, grilled veal and various tapas. Flamenco dancers, banjo players, and plenty of sangria created a festive beginning for the conference.

The main conference was preceded by an imaging consortium on Alzheimer's disease (AD). Using a wide variety of methods, investigators showed correlations between AD or aging and different imaging measures. There was particular interest in white matter lesions, which several scientists noted were closely correlated with AD. Frank-Erik De Leeuw from UMC-Stradboud in the Netherlands reported that the extent of white matter lesions correlates with medial temporal atrophy in late-onset but not early-onset AD, suggesting that late-onset and early-onset AD may have some different pathological pathways [1]. There were also several reports on brain PET imaging using the Pittsburgh compound B (PiB). William Klunk from the University of Pittsburgh estimated that over 500 patients at 12 institutions have undergone PET scans using PiB, which appears to be a reliable marker of amyloid deposition [2]. It is hoped that imaging will identify patients at risk for AD and differentiate patients with amyloid-β deposition from patients with other types of pathology.

Although there were hundreds of presentations at ICAD, a few stood out because of their importance and novelty. Perhaps the biggest news came from two independent groups of researchers at the Mayo Clinic and the University of Antwerp in Belgium. Both groups found mutations in the gene encoding progranulin that cause a form of frontotemporal dementia (FTD), the second most common form of dementia in individuals younger than 65 years old [3,4]. Since 1998, when mutations in the microtubule-associated protein tau (*MAPT*) gene on chromosome 17 were found to cause FTD with parkinsonism, geneticists have been stymied by families with FTD linked to the same region on chromosome 17 but having no *MAPT *mutations. It seemed likely that difficult-to-find mutations in *MAPT *accounted for these FTD cases, but the mutations could not be found despite heroic efforts. Geneticists then sequenced genes near *MAPT *and were rewarded by finding mutations in the progranulin gene (*PGRN*), which is located a mere 1.7 Mb away from *MAPT*. In one patient series, *PGRN *mutations accounted for at least 11% of all FTD cases and 26% of familial FTD cases. Interestingly, all the *PRGN *mutations that were reported are dominant and effectively create null alleles, meaning that the FTD phenotype results from decreased levels of progranulin in affected individuals. Additionally, the ubiquitin-positive inclusions in the brains of affected individuals do not stain with anti-progranulin antibodies, leaving the identity of the ubiquitinated protein an intriguing mystery. The discovery of *PGRN *mutations is a major advance in our understanding of FTD and will undoubtedly inspire intense research to discover the mechanism by which progranulin deficiency leads to FTD.

Another interesting story from the conference involved BACE1, the major β-secretase in the brain. Sequential cleavage of amyloid precursor protein by β-secretase and then γ-secretase produces the amyloid β-peptide (Aβ). Since inhibition of β-secretase should decrease Aβ levels in the brain, it is an attractive therapeutic target. In fact, Martin Citron from Amgen reported that over 100 patents have been filed for BACE1 inhibitors [5]. However, inhibition of BACE1 could have negative consequences if other molecules that are physiologically important require cleavage by BACE1. Christian Haass and his colleagues in Germany were attempting to find normal substrates for BACE1 and began with two key observations: 1. BACE1 expression is high post-natally but low in adults and 2. BACE1 is not expressed by invertebrates. From these clues, Haass and his colleagues thought that BACE1 may be involved in myelination, which is prominent in the post-natal time interval and is not present in invertebrates. Their hypothesis was correct: electron microscopic examination of the peripheral nerves from BACE1 deficient mice showed pronounced hypomyelination [6]. Interestingly, this hypomyelination phenotype was similar to that of neuregulin-1 haploinsufficient mice, which led Haass and his colleagues to hypothesize that neuregulin-1 was a substrate for BACE1. Next they found that neuregulin-1 levels are elevated in the brains of *Bace1*^-/- ^mice, most likely because BACE1 is the major enzyme that cleaves neuregulin-1. Finally, they demonstrated that neuregulin-1 is cleaved by BACE1 *in vitro *[7]. It is unknown whether inhibition of neuregulin-1 cleavage in adults, as could occur with the therapeutic usage of BACE1 inhibitors, would create adverse effects. Since myelination primarily occurs in childhood, decreased neuregulin-1 cleavage in older adults may not be an issue, but this remains to be proven. Overall, this was a remarkable story because the investigators deduced such a large amount of information from two seemingly small observations.

An area that has not been previously emphasized, the interaction between AD and metabolic syndromes such as obesity and diabetes, was discussed in multiple sessions. Miia Kivipelto from the Stockholm Gerontology Research Center presented epidemiological data showing that obesity, hypercholesterolemia and hypertension were all risk factors for AD and that when all three factors were present in the same individual the risk of AD increased 6-fold [8]. Other investigators found links between diabetes, peripheral insulin resistance, cerebral glucose metabolism and cognitive dysfunction, but the mechanisms involved were not clear. However, a possible connection between AD and metabolic syndromes has important therapeutic implications. Previous studies have shown that intravenous (IV) insulin administration improves cognition in AD patients, but IV insulin is not a viable therapeutic option due to the risk of hypoglycemia. To circumvent the risk of hypoglycemia and deliver insulin to the brain, Suzanne Craft from the University of Washington treated subjects with either early AD or mild cognitive impairment with daily intranasal insulin or placebo. After 21 days, the patients treated with intranasal insulin had better memory-related scores [9]. Another group, from GlaxoSmithKline, also performed a clinical trial of a diabetes-related drug. They treated 511 AD subjects with the type-2 diabetes drug rosiglitazone or placebo for 24 weeks, but found no clinical improvement on several different tests [10]. Clearly, further studies are required to determine how AD is connected to metabolic disorders and whether drugs that are commonly used to treat metabolic diseases are beneficial to AD patients.

Finally, one other notable change in this conference was the plethora of drug company representatives marketing their formulations of acetylcholinesterase inhibitors and memantine. While scientists rarely get excited about increasing numbers of drug salespeople, their presence was evidence that therapeutics the Alzheimer's research and pharmaceutical community has been developing for years are finally coming to the market. Hopefully the therapeutics being marketed at the 2008 ICAD conference in Chicago will be more numerous and effective.

## Competing interests

The author(s) declares that she has no competing interests.
